# Proprioceptive acuity into knee hypermobile range in children with Joint Hypermobility Syndrome

**DOI:** 10.1186/1546-0096-12-40

**Published:** 2014-09-08

**Authors:** Verity Pacey, Roger D Adams, Louise Tofts, Craig F Munns, Leslie L Nicholson

**Affiliations:** Physiotherapy Department, The Children’s Hospital at Westmead, Sydney, Australia; Kids Rehab, The Children’s Hospital at Westmead, Sydney, Australia; Department of Endocrinology, The Children’s Hospital at Westmead, Sydney, Australia; Discipline of Biomedical Sciences, The University of Sydney, Sydney, Australia; Discipline of Physiotherapy, The University of Sydney, Sydney, Australia; Discipline of Paediatrics and Child Health, The University of Sydney, Sydney, Australia

**Keywords:** Hypermobility, Knee, Proprioception, Hyperextension, Joint hypermobility syndrome, Ehlers-Danlos syndrome, Range of motion

## Abstract

**Background:**

Children with Joint Hypermobility Syndrome (JHS) have reduced knee joint proprioceptive acuity compared to peers. Altered proprioception at end of range in individuals with JHS is hypothesised to contribute to recurrent joint injuries and instability. This study aims to provide the first objective comparison of functional knee joint proprioceptive acuity in hyperextension range compared to early flexion range in children with JHS.

**Methods:**

Active, weight-bearing knee joint proprioceptive acuity in both hyperextension and early flexion range was tested with a purpose-built device. Proprioceptive acuity was measured using the psychophysical method of constant stimuli to determine ability to discriminate between the extents of paired active movements made to physical stops. The smallest difference in knee range of motion that the child is able to correctly judge on at least 75% of occasions, the Just Noticeable Difference (JND), was calculated using Probit analysis. Knee pain, muscle strength, amount of physical activity and patient demographic data were collected.

**Results:**

Twenty children aged 8–16 years with JHS and hypermobile knees participated. Eleven children demonstrated better proprioceptive acuity in flexion, and 9 in hyperextension (z = 0.45, p = 0.63). Matched pairs t-test found no significant difference in children’s ability to discriminate between the same extents of movement in the hyperextension or flexion directions (mean JND difference 0.11°, 95% CI -0.26° - 0.47°, p = 0.545). However, 3 children could not discriminate movements in hyperextension better than chance. Proprioceptive acuity scores were positively correlated between the two directions of movement (r = 0.55, p = 0.02), with no significant correlations found between proprioceptive acuity and age, degree of hypermobility, muscle strength, pain level, amount of physical activity or body mass index centile (r = -0.35 to -0.03, all p ≥ 0.13).

**Conclusion:**

For a group of children with JHS involving hypermobile knees, there was no significant difference between knee joint proprioceptive acuity in early flexion and in hypermobile range when measured by a functional, active, weight-bearing test. Therefore, when implementing a proprioceptive training programme, clinicians should focus training throughout knee range, including into hyperextension. Further research is needed to determine factors contributing to pain and instability in hypermobile range.

## Background

Joint Hypermobility Syndrome (JHS) is characterised by chronic joint pain, instability and multi-system involvement. Following the exclusion of other heritable disorders of connective tissue, JHS is diagnosed using the Brighton criteria, as yet not validated in children [1]. While the exact cause of pain and disability associated with JHS is unknown, reduced joint proprioception with associated sub-optimal motion control in the hypermobile range is hypothesised to be a contributing factor to symptoms associated with JHS, including poor motor co-ordination [2]. The approach to physiotherapy management of individuals with JHS published over the last 15 years has altered from advice to not move hypermobile joints into end-range [3], to advice to progress exercises so as to develop joint control in the hypermobile range, because this is where the joint is considered to be “less stable due to muscle weakness and altered proprioception” (p. e9) [4]. While this treatment paradigm of exercising into the hypermobile range is the current expert recommendation, there have been no published reports investigating proprioceptive acuity in the hypermobile range in individuals with JHS.

A number of studies have assessed knee joint proprioceptive acuity of individuals with JHS in their non-hypermobile range. A recent systematic review concluded that individuals with JHS have reduced knee joint proprioception, assessed by passive threshold to detection of movement and joint position sense error, when compared to non-hypermobile controls [2]. In the only published study investigating proprioception in children with JHS, both knee joint position sense and kinaesthesia, assessed passively with the knee flexed and not weight-bearing, were found to be significantly worse in children with JHS than in non-hypermobile controls [5].

Knee joint proprioceptive acuity of non-hypermobile adults improves as the joint moves from mid-range flexion towards the end of range of movement of extension [6]. This improvement in knee joint proprioceptive acuity towards end of range of movement is not evident in adults with JHS [6]. Similarly, children with JHS demonstrate small to no improvement in their absolute angular error when moving from a flexed, to an almost neutral knee extension position, while their non-hypermobile peers demonstrate a substantial improvement in joint position sense error moving from 25° to 10° knee flexion [5]. This suggests the usual increase in proprioceptive acuity seen towards end of range is not present in adults or children with JHS.

Therefore, to assess the hypothesised proprioceptive deficit in hypermobile range in children with JHS, our study aims to compare knee joint proprioception with the same test in both hyperextension and flexion, using a functional active proprioceptive acuity assessment technique. Results of this study can then be used to guide the knee joint range in which clinicians recommend proprioceptive training for children with JHS.

## Methods

### Patients

Children aged 8–16 years with JHS, diagnosed using the Brighton criteria, and with knee hyperextension >10°, were recruited through specialists and physiotherapists at The Children’s Hospital at Westmead, Sydney, Australia. All children met the major Brighton criterion of a Beighton score ≥4/9. Exclusion criteria were significant learning disabilities which would preclude the child from understanding the task and responding reliably during testing, or the presence of an acute lower limb injury sustained within the last 3 months, or previous knee joint surgery, any of which may result in altered proprioceptive acuity unrelated to the child’s diagnosis of JHS. The study was approved by The Children’s Hospital at Westmead and The University of Sydney’s Human Ethics Committees. Written informed consent was obtained from the guardian of the patient for publication of their individual details and accompanying images in this manuscript. The consent form is held by the authors and is available for review by the Editors-in-Chief.

Immediately prior to testing, all participants recorded their current knee pain on a visual analogue scale (VAS). Height and weight were recorded and body mass index (BMI) calculated. Height, weight and BMI centiles were then calculated from age and gender-specific reference values [7]. Joint hypermobility was confirmed using the Beighton score [1].

Quadriceps and hamstrings isometric muscle strength at neutral (0°) knee extension was measured in a side-lying position using a hand-held dynamometer. Three measures were taken on the side on which proprioception was assessed and the maximum measure obtained used.

The Adolescent Physical Activity Recall Questionnaire [8] was completed by all participants. This questionnaire collates the frequency and duration of all organised and non-organised physical activity undertaken, and has demonstrated acceptable reliability and validity in adolescents [8]. From the information obtained the average number of minutes per week spent performing moderate and vigorous activities [9] was calculated.

An active, weight-bearing technique was employed for testing knee joint proprioception, to provide information about a child’s proprioceptive acuity relevant to functional weight-bearing tasks. Proprioceptive information comes from muscles, joint capsule, ligaments, menisci and subchondral bone of joints [10], all of which are activated in weightbearing activities.

The psychophysical method of constant stimuli [11, 12] was used to generate a Just Noticeable Difference (JND) as the knee proprioceptive sensitivity measure. This method has demonstrated excellent test-retest reliability in adults [13] and is able to discriminate between individuals with and without knee injuries and post rehabilitation [14]. Reliability in children has not been tested.

**Figure 1:**
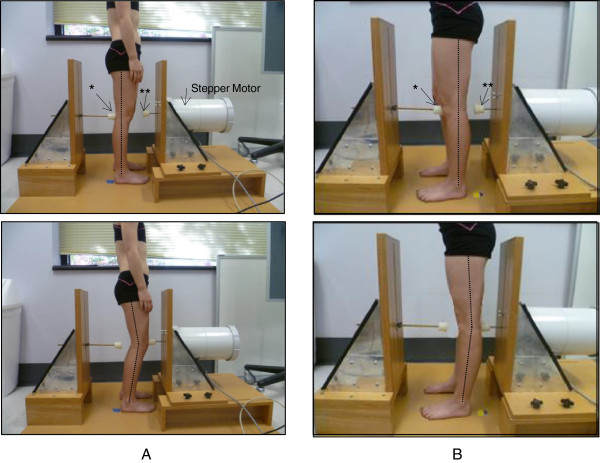
**The purpose-built device and participant set-up for testing of proprioceptive acuity.** *represents the fixed stopper. **represents the moveable stopper driven by the stepper motor (inside the casing). **A**. Testing in early flexion range. **B**. Testing in hyperextension range.

One knee, randomly chosen, was tested in two different ranges of movement, 0-10° knee flexion and 0-10° of available hyperextension, using a purpose-built device (Figure 1). Each participant stood with their knee at neutral extension, touching the fixed stopper. The device was screened to occlude vision and clues as to the extent of movement permitted. Randomisation determined the order in which flexion and hyperextension were tested. Proprioceptive acuity in flexion was tested with the child instructed to move from neutral knee extension with the back of their knee touching the immovable stopper, flexing (descending) until the front of their knee contacted the moveable stopper which was driven by a stepper motor to randomly place it in one of five positions. Proprioceptive acuity into hyperextension was tested separately with the participants instructed to move from the two-legged squat position with the front of their knee contacting the immoveable stopper, extending (ascending) until the back of their knee contacted the moveable stopper. Each pair of movements included one movement to the mid position of the set (the standard stimulus set 3 cm from the starting neutral knee position and = 8.1° of flexion or hyperextension) and one movement made to one of the other four positions (the variable stimulus). The difference in distance between each stimulus (2.04 mm = 0.56°) was determined during pilot testing to create a pairwise discrimination task that on each trial would be difficult but still possible for most participants to discriminate. The pairs of movements incorporating the variable stimuli furthest away from the standard stimulus were mostly discriminable even by those participants with poor knee joint proprioceptive acuity, while the pairs of movements including the two variable stimuli closest to the standard were more difficult for all participants to discriminate. On each trial, after making the two movements and returning to the start position, the participant was instructed to report whether the second movement was bigger or smaller than the first. Participants performed 56 pairs of movements (112 movements in total, with 14 sets of 4 paired comparisons incorporating each variable stimulus) presented in a random sequence. Testing took a maximum of one hour. Participants could rest at any stage if they were tiring or had difficulties in maintaining concentration.

### Statistical analyses

Data analysis was performed using SPSS v 21. Descriptive statistics, including means, standard deviations (SDs), ranges and frequencies, were used to summarize the participants’ characteristics.

The psychophysical method of constant stimuli was used to estimate the JND. The JND is the smallest difference in knee range of motion that the child is able to correctly judge on at least 75% of occasions. A JND of 0.5° of knee active movement has previously been found to be the mean difference between elite and novice sporting participants [15] and was therefore hypothesised to be an appropriate expected difference in proprioceptive acuity between the flexion and hyperextension range. To have 85% power for an effect size of a 0.5° JND at 5% significance, 20 participants were required.

Proprioceptive acuity data were analysed using Probit analysis, which calculates the parameters of the best-fitting cumulative normal ogive for each participant, thereby providing the JND in millimetres. This was then converted, using trigonometry, to a JND in degrees to allow clinical interpretation of the proprioceptive accuracy data. When participants performed at or below the level of chance (i.e. ≤28/56 correct responses), the JND was not able to be estimated.

For participants for whom a result in each direction could be calculated, the mean and SD of the flexion JND and hyperextension JND were calculated and compared using a matched pairs t-test. With all 20 children considered, the proportion of children whose acuity was greater in flexion was tested against the null hypothesis of 0.5 (i.e. chance – children equally likely to be better in hyperextension than in flexion) using the z-test for a proportion. The strength and direction of the linear relationship between the participant characteristics of age, BMI centile, pain, maximum muscle strength and amount of physical activity participation (continuous data) and knee joint proprioceptive acuity in flexion and extension were examined using Pearson’s correlation coefficient, with r = 0.1 considered as weakly, r = 0.3 as moderately and r ≥ 0.5 strongly correlated [16]. To determine if a difference in proprioceptive acuity exists between boys and girls (binary participant characteristic), an independent groups t-test was performed.

## Results

Twenty children (14 girls, 6 boys) with JHS (including hypermobile knees) participated in this study. Six children reported a history of chronic knee pain and instability, three reported a history of chronic knee pain only, three reported recurrent knee instability only, with eight reporting no previous knee symptoms despite their hypermobility at this joint. To meet the Brighton criteria for a diagnosis of JHS, 17 children fulfilled both major criteria (Beighton score ≥4/9 and chronic pain in ≥4 joints), with the remaining 3 children fulfilling 1 major (Beighton score ≥4/9) and 2 minor criteria (chronic pain in 1–3 joints, recurrent joint dislocations and/or ≥3 soft tissue injuries).

Individual and mean participant characteristics and proprioceptive acuity scores are presented in Table 1 and Table 2. Four of the 20 participants required a rest during testing. The JND of 3 participants could not be calculated as they had performed at or below the level of chance during testing. With 17 participants for whom a JND could be calculated in each direction, the study had 80% power to detect differences in proprioceptive acuity in flexion and hyperextension directions as large as 0.48° at the 5% significance level. There was no significant difference between the flexion and hyperextension JNDs of these 17 participants (mean difference 0.11°, 95% CI -0.26° - 0.47°, p = 0.55). Overall, 11 participants had better (lower JND) proprioceptive acuity into flexion than hyperextension, while nine performed better into the hyperextension range (z = 0.45, p = 0.63).

**Table 1:** **Individual proprioceptive acuity scores and participant characteristics**

Study no	Gender	Age	Pain/10	Beighton/9	Sports participation	HE/56 (%) correct	F /56 (%) correct	HE JND°	F JND°	Difference (HE-F JND)
20	F	14	7.5	9	Dance, Badminton, Rugby union, Soccer	42 (75)	37 (66)	0.30	0.45	-0.15
18	F	9.5	0	9	Nil	41 (73)	36 (64)	0.32	0.61	-0.29
3*	F	16.7	0.5	5	Athletics, Netball	37 (66)	37 (66)	0.51	0.47	+0.04
17	F	14.4	0	8	Dance	33 (59)	34 (61)	0.66	0.78	-0.12
14	M	11	0	8	Athletics, Dance, Gymnastics	35 (63)	30 (54)	0.66	1.34	-0.68
9	F	10.2	5.3	4	Softball, Hockey	37 (66)	30 (54)	0.67	1.15	-0.48
11	F	14.2	6.9	9	Trampolining, Dance	34 (61)	37 (66)	0.71	0.38	+0.33
7	F	10.4	2.3	8	Nil	35 (65)	30 (54)	0.71	1.57	-0.86
1	F	8.2	0	9	Dance	32 (57)	38 (68)	0.80	0.45	+0.35
2	F	10.3	0	7	Athletics, Swimming, Soccer, Cycling, Dance	37 (66)	33 (59)	0.80	1.06	-0.26
10	F	13.6	4.5	6	Softball, Hockey, Swimming	34 (61)	32 (63)	0.81	1.15	-0.34
15	M	10.5	0	8	Basketball, Hockey, Australian football, Soccer, Bushwalking, Table tennis, Soccer	34 (61)	34 (61)	1.14	0.73	+0.41
5*	M	9.9	0	7	Tennis, Soccer, Cricket	33 (59)	33 (59)	1.16	0.99	+0.17
19	F	12.8	0	8	Tennis, Netball, Dance, Cycling, Bushwalking	34 (61)	30 (54)	1.34	1.63	-0.29
12	F	9.9	0	8	Swimming, Trampolining, Netball	30 (54)	40 (71)	1.61	0.40	+1.21
6*	M	15.5	2.4	6	Martial Arts, Cycling	32 (57)	35 (63)	1.63	0.81	+0.82
13	M	9.1	0	8	Dance	29 (52)	31 (55)	3.98	2.02	+1.96
8	F	11.2	1.8	6	Swimming, Tennis, Dance, Pilates	28 (50)	35 (63)	NA	0.72	+NA
4	F	11.2	3.4	8	Dance	27 (48)	32 (57)	NA	0.88	+NA
16*	M	11	4.2	8	Swimming, Australian football, Soccer	28 (50)	35 (63)	NA	0.72	+NA
Mean (SD)		11.69 (2.32)	1.94 (2.52)	7.45 (1.4)		33.6/56 (60%) (SD 4.04)	33.95/56 (61%) (SD 2.98)	1.05 (0.85)	0.92 (0.45)	0.11 (0.71)

Smaller JND indicates better proprioceptive acuity.

HE-F: -ive number indicates proprioception in hyperextension range is better than in flexion range, +ive number indicates proprioception in flexion range is better than in hyperextension range.

NA indicates that the JND was not able to be attained (i.e. ≤28/56 correct responses).

*indicates participant required rest during testing.

**Table 2:** **Participant characteristics**

Participant characteristic	Mean (SD)	Range
BMI centile	50.89 (32.67)	3.34 – 98.06
Maximum quadriceps muscle strength (N)	6.28 (2.83)	2.3 – 12.3
Maximum hamstrings muscle strength	5.49 (2.94)	2.0 – 11.3
Hours per week of moderate – vigorous physical activity	7.46 (3.79)	1.0 – 17.55

There was a strong and significant correlation between proprioceptive acuity into flexion and hyperextension range (r = 0.55, p = 0.02) indicating those who performed well in one direction, also performed well in the other direction. There was no statistically significant correlation between proprioceptive acuity in either direction and the participant's Beighton score, BMI centile, knee pain immediately prior to testing, maximum quadriceps muscle strength, maximum hamstrings muscle strength, minutes spent undertaking physical activity, or age (flexion: r = -0.35 - -0.18, df = 18, all p ≥ 0.13; hyperextension: r = -0.27 - -0.01, df = 15, all p ≥ 0.25). When comparing the proprioceptive acuity between boys and girls in either flexion (mean difference 0.27°, 95% CI -0.28° – 0.81°) or hyperextension (mean difference 0.95°, 95% CI -0.67° – 2.56°) it can be seen that both confidence intervals cross zero.

## Discussion

In a group of twenty children with JHS and knee hypermobility tested for knee joint proprioceptive acuity, nine children had better acuity scores in hyperextension, and eleven had better acuity in flexion. Because the children could not see their knee movements, and judging movement extent was therefore based solely on proprioception, these data suggest that active, weight-bearing knee joint proprioceptive acuity is as accurate when children with JHS move into hyperextension as it is when they move into flexion.

Functional activities of daily living require a child to be upright and weight-bearing, with joint pain often reportedly exacerbated following activity. It is unknown if children with JHS demonstrate knee hyperextension during many functional activities, however gait analysis shows that children with JHS utilise less knee flexion during walking than non-hypermobile children, exhibiting knee hyperextension during stance phase [17]. Therefore, even if advised to avoid the hypermobile range, it is likely a child with JHS will inadvertently use their knee joint hyperextension when walking. If the treating clinician incorporates proprioceptive training within a treatment programme for a child with JHS, this training should not be limited only to neutral knee extension. Given the findings from this study demonstrate that proprioceptive acuity is equal in both hyperextension and flexion joint ranges, and children with JHS do hyperextend during gait [17], improvement in proprioceptive acuity in both directions is equally required for use in functional tasks.

However, the apparent lack of control in hyperextension, commonly seen in children with JHS, is unlikely to be solely attributable to impaired proprioception. In a recent randomised controlled trial, exercises targeting eccentric hamstring control towards and into hypermobile range demonstrated significant improvements in knee pain in children with JHS [18]. Further research is required to understand other contributing factors to poor joint control in the hyperextension range. In particular, assessment of the role of the hamstrings in knee extension control, and comparison of active, weight-bearing knee joint proprioceptive acuity of children with JHS with their non-affected peers, are warranted.

Evidence currently suggests that proprioceptive acuity is use-dependent. This has been demonstrated by elite athletes showing better proprioceptive acuity than novices [15] and that improvement obtained from proprioceptive training is joint specific in adults [19]. In the current study, a JND in the hyperextension range could not be estimated for three of the 20 participants, indicating that some children with JHS have a very low level of proprioceptive acuity in the hypermobile range. Further research in this population investigating proprioceptive acuity, pain, level of physical activity performed (amateur, elite), use of knee hyperextension during sports and gait parameters may provide further insight as to how children with JHS and poor hyperextension proprioceptive acuity may differ from their peers with JHS with better acuity scores. One possible contributing factor may be kinaesiophobia and further investigation of whether this is associated with reduced proprioceptive acuity in these children is warranted.

Proprioceptive acuity scores in early flexion and hyperextension range were moderately/highly and significantly correlated in this study. This correlation between proprioceptive acuity in different movement directions has been also been demonstrated at the ankle [11]. No other significant correlations were found suggesting that pain at the time of testing and muscle strength deficits presenting as part of JHS in childhood did not alter the child’s ability to undertake an active proprioception test. Pain at the time of testing was recorded to ensure the active, weight-bearing technique of proprioceptive acuity testing did not exacerbate pain or alter the child’s ability to undertake the test. Further assessment of the presence and extent of the child’s pain within their regular daily activities may provide further insight into factors affecting each child’s proprioceptive acuity.

The findings of this study further support the evidence suggesting that proprioceptive acuity scores of athletes are unrelated to their amount of sport-specific practice [20]. The measures collected here were included in correlations to check on whether proprioceptive acuity can be deemed a separate attribute to the amount of physical activity performed and to ensure that the known muscle weakness [5] and pain [21] experienced by children with JHS was not impacting upon their proprioceptive ability. Furthermore, the lack of correlation between age and proprioceptive acuity scores suggests that children 8 years and over have the attention and cognitive ability to undertake an active test using psychophysical methods, allowing for functional assessment of proprioception. Rests were allowed during testing to accommodate for any children with joint pain, fatigue or difficulties maintaining concentration on the task. Children with JHS are known to require more rests than their healthy peers [15], and four of the 20 children participating in this study required a rest during testing, which may have affected their results in comparison to their peers (Table 1). Because the rest breaks during testing were taken equally often in flexion and hyperextension testing, with two children taking a rest in each condition, rest therefore had no systematic effect on the comparison between proprioceptive sensitivity for these two directions of knee movement.

## Conclusion

Functional, active, weight-bearing testing of proprioception can be successfully performed in symptomatic children from 8 years, providing valid information to guide clinicians in management. Children with JHS demonstrate no alteration in their proprioceptive acuity in the hypermobile range compared to early flexion range of knee movement, and any proprioceptive training programme should therefore, be performed throughout full range, including this early hypermobile range. By performing these exercises through full hypermobile range, physiotherapists do not need to provide children with close supervision to prevent movement into hyperextension, or provide physical stops to knee hyperextension, such as bracing or taping, when undertaking proprioceptive exercises. Further research is required to understand the contributing factors to pain and instability in the hyperextension range.

## Abbreviations

JHS: Joint hypermobility syndrome
JND: Just noticeable difference
VAS: Visual analogue scale.
